# Parental Practices of Controlling and Supporting the Autonomy of Elementary School Children and Early Adolescents in Russia: A Qualitative Study

**DOI:** 10.11621/pir.2024.0201

**Published:** 2024-06-01

**Authors:** Anastasia K. Nisskaya, Ekaterina M. Tsyganova

**Affiliations:** a *HSE University, Moscow, Russia*

**Keywords:** parental practices, autonomy, independence, volitional functioning, autonomy support, autonomy control

## Abstract

**Background.:**

Children’s and adolescents’ development of autonomy depends on the relationship with their parents and the parents’ child-rearing practices. These might be aimed towards supporting or restricting autonomy, as well as its different aspects, such as independence or volitional functioning.

**Objective.:**

To compare the practices described by foreign researchers as being the most beneficial for supporting autonomy with those used on a daily basis by Russian parents of primary school children and early adolescents.

**Design.:**

We conducted 26 semi-structured interviews with 16 mothers and 10 fathers of primary school children (n = 10) and early adolescents (n = 16).

**Results.:**

The practices of autonomy support and control used by parents were mostly similar to those described in foreign literature. However, new features were found: Guidance, Explanation of Patterns, and “Area of Responsibility”. The behavior of Russian parents can be described through practices specific to different situations. Qualitative research suggests the absence of a unified style of behavior in relation to children’s independence. Two types of autonomy support practices were used: encouraging independence and support for volitional functioning. Encouraging children’s volitional functioning was perceived by parents as something that guides their behavior, yet mention of this practice was much less explicit than mention of encouraging independence.

**Conclusion.:**

Further reflection is required on the observed situationality of practices – whether it should be assessed as chaotic, hindering autonomy, or flexible, promoting it.

## Introduction

Numerous scholars have studied parental actions regarding children’s autonomy (Kouros & Garber, 2014; Soenens et al., 2007; Van der Kaap-Deeder et al., 2017; Won & Yu, 2018). Their findings indicate that the relationship with parents and their methods of child-rearing play a crucial role in the development of autonomy in children and adolescents.

### Two Aspects of Autonomy

Children’s autonomy could be defined through two dimensions: “independence” and “volitional functioning” (Soenens et al., 2017). The first, technical, aspect is separation from adults and comes from the separation-individuation theory proposed by M. Mahler (Blos, 1967). The second aspect, goal-setting according to one’s own desires, is based on self-determination theory (Ryan & Deci, 2000). These desires are correlated with basic psychological needs.

Two aspects of autonomy are also presented in the Russian approaches. The internalization of action (becoming independent in its performance) and starting a cooperative action with adults to develop this kind of action are regarded as two categories of autonomy in the Russian cultural-historical approach (Tsukerman & Elizarova, 1990). The foreign age-psychological approach also acknowledges two sides of autonomy: value components of action, taking responsibility for it in addition to independent behavior (Molchanov et al., 2017). However, the Russian approach is different in its objective, since it focuses on the process of establishing autonomy rather than on its outcomes, which are the focus of the foreign approach.

### Parental Actions Regarding Children’s Autonomy

Parental actions fostering and controlling autonomy could be defined differently depending on the approach. The first one is the “parenting styles” approach. Parenting styles are understood as typical, consistent types of parental interactions with a child. Among them, the most defined are: authoritarian, permissive, and authoritative (Baumrind, 1966). Intermediate, mixed variants of these styles are also discussed (Vasiou et al., 2023). Although the “parenting styles” approach is considered outdated nowadays because of the impossibility of capturing the full repertoire of situational fluctuations of parental behavior (Smetana, 2017), it is still broadly used in contemporary studies as it provides a clear definition of parental behavior (e.g., Niu et al., 2023; Vasiou et al., 2023).

In comparison, there is the approach of “parental practices”, where contextual actions with a child are distinguished.

**Promoting autonomy.** There are several practices that are considered beneficial for autonomy: two types of autonomy support as well as attending to a child’s other needs (competence and relatedness) and providing structure.

**Autonomy support** is manifested through supporting the child’s initiative, providing freedom to solve problems independently that are within the child’s capability, and being willing to look at a situation from the child’s perspective (Grolnick & Pomerantz, 2009). The parent also responds empathically to the child’s feelings and expressions, provides opportunities for choice and self-expression, and explains the norms and reasons for limitations (Joussemet et al., 2008).

Autonomy support might be differentiated by types of autonomy promotion. Teaching rules, loosening control, and increasing emotional distance helps to ensure *independence*, while explaining the importance of norms and creating conditions for their integration supports *volitional functioning* (Soenens et al., 2007). Parents who support independence encourage the child’s independent decisions in the same way as the parents who support volitional functioning. However, in the latter case, the parent actively accompanies the child in this process, letting go gradually, and fosters the formation of choices that are consistent with the child’s interests.

Autonomy support promotes creativity (Armour et al., 2022), perseverance, long-term goal setting (Du et al., 2023), intrinsic goal setting, and intrinsic learning motivation (Ahn et al., 2023; Froiland, 2011). It is negatively associated with externalized problem behaviors in children (Feng & Lan, 2020) and positively with their psychological well-being (Vasquez et al., 2016). In the long term, autonomy support is associated with high levels of children’s autonomy and competence and low levels of career indecision (Ahn et al., 2023).

**Attending to a child’s other needs** occurs as parents acknowledge the suffciency of the child’s skills and abilities (competence), attend to the child, and provide opportunities for caring for others (relatedness) (Ryan & Deci, 2000). To do this, parents can use practices such as providing a variety of choices, recognizing the importance of a point of view, explaining if choices are limited, providing opportunities to care for another, providing nurturing, providing space, and creating situations of success. Doing so with a positive attitude may ensure the child’s self-efficacy in learning and positive affect (Moè & Katz, 2018).

Parenting that promotes relatedness acts as a protective factor against an inability to regulate one’s own emotions and behavior (Rothenberg et al., 2020), whereas practices that promote competence have a positive effect on children’s academic performance in reading and mathematics in the future (Puccioni, 2018).

**Providing structure.** Grolnick and Pomerantz (2009) have proposed a distinction between strict control-domination, on the one hand, and restricting a child’s autonomy through guidance and organizing the environment with clear rules and prohibitions, on the other. The latter they call “structure”, which can be understood as consistent behavioral control through the provision of clear feedback, rules and norms understandable to the child (Farkas & Grolnick, 2008).

Setting structure, supporting needs, and promoting autonomy together are beneficial for the academic motivation of children and their self-regulated learning (Farooq & Asim, 2020). However, provision of structure and attending needs without autonomy support are not as effective as with it and cannot compensate for its absence (Hornstra et al., 2021).

### Controlling Autonomy

**Behavioral control** is about regulation of children’s behavior, pastimes, and whereabouts (Grolnick & Pomerantz, 2009). It allows setting a frame of reference to ensure that the environment is predictable for the child.

Parental behavioral control usually has beneficial effects: it positively affects adolescents’ creativity and autonomous motivation (Chen et al., 2021). When psychological control is at a higher level, behavioral control might reduce the negative impact on adolescent life satisfaction (Leung & Shek, 2020). Some studies show that in conjunction with warmth, it predicts rule-breaking (at age 9) and aggression (at age 10) across cultures (Rothenberg et al., 2020). However, we assume that these results do not contradict the existence of child autonomy in indulging in these behaviors.

It might be diffcult to distinguish between behavioral control and setting structure, as both restrict a child’s autonomy by setting rules for their behavior, but because these practices only establish a comprehensible set of guidelines, they do not contradict children’s needs. Therefore, while examining empirical data, we evaluated behavioral control and structure together as practices that support autonomy.

**Psychological control** defines a parent using manipulative strategies such as inducing guilt or shame, undermining the child’s point of view, and withdrawing love (Soenens & Vansteenkiste, 2010). Through psychological control, a parent may be guided by his or her position in decision making and disregard the child’s opinion, preventing the child from solving his or her own problems (Froiland, 2011).

Adolescents’ psychological health, problematic behavior, emotion regulation, and academic performance correlate negatively with parental psychological control (Fang et al., 2021; Gao et al., 2021; Yan et al., 2020).

**Table 1:** Practices of Controlling Autonomy and Promoting Autonomy

Promoting autonomy	Controlling autonomy
**Meeting the needs of the child** Providing a variety of choices Explaining limited choices Recognizing the importance of the child’s point of view Caring (for the child and enabling the child to care for someone else) Providing space for initiative	**Psychological (internal) control** Guilt induction and manipulation Provoking anxiety Devaluation of the child’s viewpoint Insensitivity to the child’s needs
**Structure and behavioral (external) control** Prohibitions and requirements Sanctions and penalties Deadlines Rewards Clear rules (prohibitions, requirements) Help with tasks and self-organization Help in decision making Communication of confidence in the child’s competence	

In Russian scholarly works, psychologically controlling practices and violence are presented as undermining autonomy, whereas promotion of autonomy and structuring the child’s behavior are considered beneficial for autonomy development (Koroleva, 2023; Polivanova et al., 2020).

### The Problem with Applying the “Parental Practices” Approach to Russian Parents

Several diffculties might arise in applying the “parental practices” approach to Russian parents.

First, even while the term “parental practices” seems more accurate than “parenting styles”, there are issues with identifying these practices, because they are not as readily apparent as “parenting styles” and overlap in many areas. Also, the fact that the practices mentioned above are based on theoretical presumptions rather than real-world parenting scenarios makes it diffcult to categorize situations in which to apply them. Given that independence can be more easily distinguished in real-world situations, autonomy might be frequently comprehended and fostered in terms of independence.

Second, it is not obvious how these practices would manifest in everyday life, particularly when it comes to Russian samples. Parents may use practices that are considered as culturally normative and beliefs about the legitimacy of their actions can shape their practices and contribute to a sense of self-efficacy (Lansford, 2022). There is evidence that autonomy support varies across cultures (Benito-Gomez et al., 2020; Marbell-Pierre et al., 2019) and behavioral control is culturally specific in its effects (not in all countries can it lead to an increase in externalization and internalization problems in children) (Rothenberg et al., 2020). Russian parents, for example, compared to U.S. parents, tend to be perceived as more controlling (Chirkov & Ryan, 2001) and feel the need to help children with school assignments up to grade 6, considering them not autonomous enough to cope on their own (Polivanova et al., 2023). So, we expect that Russian parents will be similar to foreign parents in the presence of constructs, yet autonomy-promoting practices, especially support for volitional functioning, will be less prominent than autonomy-controlling practices.

In addition, autonomy-supporting practices may differ depending on the age of the children, and autonomy-supporting practices with elementary school children have been relatively poorly studied (Vasquez et al., 2016).

Thus, based on the descriptions given, it is unclear how to differentiate between practices, and we may observe variations in their use in Russian parents’ daily lives. Therefore, we want to reconstruct these practices from the lives of parents in order to comprehend their contextual differences – how they vary from one another in different situations — as well as the cultural distinctiveness of the application of these practices.

So, the purpose of the study was to compare the practices described by foreign researchers as the most favorable for autonomy support with the practices used by Russian parents of primary school children and early adolescents on a daily basis.

Research questions:

1. What are the specific features in autonomy support and control among Russian parents, compared to the examples presented in the foreign literature?
2. In what situations do Russian parents support and control children’s autonomy?

We will use a deductive framework to address these issues, but we will look for additional in-vivo codes to extend the theory.

## Methods

### Participants

Informants were recruited via an online application form, which included a description of the study, terms of use of personal data, and questions about socio-demographic characteristics (name, sex, age, place of residence, age of the child, etc.), and consent to participate in the study. The pool of informants was created and then only those who met the key criteria were selected. Key conditions: mothers and fathers from different Russian cities (to balance socioeconomic state), with a child of primary school or early adolescent age. We tried to include both mothers and fathers to offset any discrepancy in the mentioned practices. Studies show that parents may differ in the warmth provided to children: mothers are predominantly more authoritative, while fathers are more authoritarian (Yaffe, 2020) and mothers might also show more autonomy support than fathers (Hughes et al., 2018).

Sixteen mothers and 10 fathers participated in the study. Some of them had two or more children, including both elementary schoolers and young adolescents; at the beginning of the interview they agreed to choose only one child for the further discussion. Ten parents described the experience of interaction with primary schoolchildren, 16 with early adolescents. Eleven informants lived in cities with a population of more than 1 million (Moscow, St. Petersburg, Chelyabinsk), 12 in large cities with a population ranging from 100,000 to 1 million (Irkutsk, Yuzhno-Sakhalinsk, Saratov), and 3 in small towns or rural areas (Kurgan region, Rostov region, Moscow region) (Table 2).

**Table 2:** Participants’ Characteristics

Number	Sex	City/Region	The child about whom the parent was talking	Age of parent	Education	Number, age and sex of children F — female M — male
1	F	Rural area, Rostov region	Primary school child	36	Higher education	2 children: 7M and 2.5F
2	F	Small town, Moscow region	Primary school child	35	Higher education	3 children: 2M, 8M, and 13M
3	M	Moscow	Primary school child	39	Higher education	2 children: 6F and 9M
4	F	Moscow	Primary school child	37	Higher education	2 children: 9M and 6F
5	F	Chelyabinsk	Primary school child	39	Higher education	2 children: 10M and 5F
6	F	Irkutsk	Primary school child	36	Higher education	2 children: 6F and 10F
7	F	Small town, Moscow region'	Primary school child	38	Higher education	2 children: 10M and 17M
8	F	Saratov	Primary school child	43	Higher education	3 children: 23M, 18F, 10F
9	F	Small town, Moscow region	Primary school child	35	Higher education	2 children: 10M and 7M
10	M	Moscow	Primary school child	35	Higher education	2 children: 6F and 3M
11	M	Moscow	Primary school child	36	Higher education	2 children: 6M and 9M
12	M	Moscow	Primary school child	42	Higher education	1 child: 6F
13	F	Small town, Moscow region	Early adolescent	45	Higher education	2 children: 19F and 11M
14	M	Moscow	Early adolescent	35	Higher education	1 child: 11F
15	F	Moscow	Early adolescent	39	Higher education	1 child: 11F
16	M	Feodosia – Moscow	Early adolescent	32	Higher education	1 child: 11F
17	F	Kazan	Early adolescent	41	Higher education	3 children: 7F, 12F, and 15M
18	F	Moscow	Early adolescent	48	Higher education	3 children: 21F, 12F, and 10F
19	F	St. Petersburg	Early adolescent	35	Higher education	1 child: 12F
20	M	Yuzhno-Sakhalinsk	Early adolescent	39	Higher education	2 children: 13M and 17M
21	M	St. Petersburg	Early adolescent	44	Higher education	1 child: 12F
22	F	Moscow	Early adolescent	39	Higher education	1 child: 13M
23	F	Rural area, Kurgan region	Early adolescent	45	Secondary vocational education	2 children: 13F and 19M
24	F	Nizhny Novgorod	Early adolescent	36	Higher education	2 children: 9M and 13F
25	M	Yuzhno-Sakhalinsk	Early adolescent	41	Higher education	4 children: 6F, 13F, 14F, 17F
26	M	Yuzhno-Sakhalinsk	Early adolescent	49	Higher education	2 children: 13M and 6M

### Procedure

Twenty-six semi-structured online interviews were conducted. The guide was developed on the basis of literature analysis and two focus groups with parents of primary schoolchildren and early adolescents. As a result of the focus group, a number of practices recommended by researchers and also used by parents were identified. Some of the practices considered beneficial for children’s independence are not used by parents. Parents talked about such practices as reliance on the individual characteristics of the child, his or her interests, individual qualities; the gradual and phased transfer of responsibility for action to the child; the need to provide the child with skills and information for independent action. The practice of mutual compliance with agreements was also used, creating a sense of success in the child, the social significance of what he is doing, and the absence of harsh sanctions for taking excessive initiative. The identified practices and ideas formed the basis of the interview guide (Appendix 1).

Interviews were transcribed by an independent person outside the research team. Deductive thematic analysis (Fereday & Muir-Cochrane, 2006) was conducted using the MAXQDA2022 program (VERBI Software, 2021).

A post-positivist paradigm was implemented: the process of conducting and analyzing the interviews was accompanied by reflection on motives and expectations of researchers in order to minimize the influence of subjectivity in interpreting the material. The joint coding was carried out by the authors so that at least 30% of the interviews completely matched the codes. See code book in Appendix 2.

## Results

### Practices of the Child’s Autonomy Control

**Psychological control.** Psychological control was found in 10 interviews and was mentioned when a child expressed a protest or requested independence and volition that were considered untimely by parents. Parents talked about inducing guilt, provoking anxiety, disregarding the child’s point of view and need for autonomy. No one mentioned practices that could be interpreted as withholding love.

***Inducing guilt and manipulation*** were found in situations where parents intended to influence the child’s behavior at school and subject the child’s behavior to social norms by enunciating certain emotions to be internalized. “...sometimes I use manipulation. I say, ‘The teacher has provided you with such trust, you have been chosen’ ” (36-year-old mother of a 7-year-old boy, rural area).

***Provoking anxiety*** was related to doubts about a child’s ability to cope with household chores, self-organization, and moving around the area or city. It was also attributed to the need to clarify the significance of the parental role. Parents generated doubts about the child’s competence, showing him or her that the challenges of taking care of oneself are too hard to overcome without parental assistance.

“He said, ‘I’ll come home by myself’. I said, ‘How?’ He said, ‘I’ll take a cab’. ‘Who’s gonna pay for it?’ ‘I’ll take a shuttle’. ‘Which one? You came home from school, what then? Will you warm up your food? Where will you warm it up, what will you warm up?’ ” (36-year-old mother of a 7-year-old boy, rural area).

**Table 3:** Frequencies of Initial Codes

Name	Definition	Frequency (number of interviews)
**1. Psychological control**		
Guilt induction and manipulation	Inducing guilt for actions or their outcome and manipulating child’s emotions	5
Provoking anxiety	Provoking child’s anxiety when he or she tries to do an action or make a choice or refrain from doing it	3
Insensitivity to the child’s need for autonomy	Denying the right to behavior chosen by the child	3
Disregarding the child’s point of view	Ridiculing or not considering the opinion of a child	2
Withholding love	Denying affection for a child	0
**2. Behavioral control and structure**		
Explicit prohibitions	Setting prohibitions	17
Punishment	When a prohibition is violated, parents impose a punishment in order to stop the unwanted behavior and deter future violations	16
Rewards	Setting rewards	7
Clear, consistent requirements and deadlines	Setting requirements and deadlines that are clear and manageable for the child	14
Helping with tasks and self-organization	Helping children with self-organization and completing tasks	20
Helping with making decisions	Helping children with making decisions	5
Conveying confidence in the child’s competence	Conveying the confidence that a child can behave well and act independently	5
**3. In vivo**		
Encouraging reflection and analysis of the situation	Parent tells child to analyze the situation and draw conclusions from it	13
Guidance	Nudging in order to change unwanted non-autonomous behavior of a child	17
Explanation of patterns	Explaining outcomes of a certain behavior	20
“Area of responsibility”	Something that a child should do without parental assistance	10
**4. Direct autonomy promotion and meeting other needs**		
Providing a variety of choices	Demonstrating various possibilities of behavior and spheres of activity	9
Explanation when choices are limited	Explanation when child’s choices are limited	12
Providing care and enabling care for another	Parents provided care for children and enabled their attempts to care for others	18
Providing space	Parent in a passive position and steps aside while giving the child freedom to do an activity or choose an option on their own	21

***Insensitivity to the child’s need for autonomy*** was expressed by denying the right to behavior chosen by the child in case of problems in learning, unwillingness to participate in a sports club or participate in extracurricular activities at school.

“The teacher asked him to do a little performance.... He dug in his heels: ‘I’m not going to do that, that’s the role of a moron’. I tried in a good way, at first I persuaded, I asked, I promised chocolates, sweets, and everything in the world. No. Then I just yelled at him. We had a fight for two and a half hours. Sometimes I was swearing, sometimes I calmed down, tried to move away, then again” (36-year-old mother of a 7-year-old boy, rural area).

***Rejection of the child’s point of view*** was described by parents if the child chose inappropriate online content (“nonsense”) and refused to attend sports activities chosen by parents.

“I’m making decisions for you.... It’s a fine line. Here you have autonomy, but when you cross this line, then no one needs your autonomy, sorry” (36-year-old mother of a 7-year-old boy, rural area).

The practices of psychological control are described relatively rarely by Russian parents. This suggests that Russian parents cannot be unambiguously described as more controlling than parents from other countries, at least with regard to psychological control.

### Practices of the Child’s Autonomy Promotion

**Behavioral control and structure.** Practices of this type were found in 25 interviews, suggesting that most parents use behavioral control and seek to set a clear framework within which the child can navigate and gain autonomy. Th ere was rarely support for children’s volitional behavior; instead, parents supported the volition of children in achieving goals in socially accepted spheres. Parental understanding of the practices sometimes differed from the theoretical one. For instance, explicit behavioral control was seen as providing space for choice.

***Explicit prohibitions*** were used by parents to regulate the amount of screen time, its content, bedtime, and participation in cyber-aggression. Parents also prohibited health-threatening behaviors: getting tattoos before age 18, unlimited consumption of sweets, breaking traffic rules, traveling to remote areas or at night, going to potentially dangerous places (rallies, abandoned houses), smoking, drinking alcohol, and using profanities at home. Parents formulated prohibitions as: “Let me make a decision for you here” (39-year-old father of a 9-year-old child, megapolis), “We have decided that you will not do that” (37-year-old mother of a 9-year-old child, megapolis).

When describing ***punishment*** practices, parents talked about restricting smartphone, computer, and television use: “... I can take away the phone, take away the TV remote, tomorrow we will rest from TV, you will only draw, walk outside, play board games” (Mother of a primary school child, large city).

Punishment sometimes took the form of monetary sanctions for missed classes or bad grades: “We have a grading system ... an A is 100 rubles, a B is 50 rubles, a C is minus 50 rubles, and a D is minus 100 rubles” (44-year-old father of a 12-year-old adolescent, megapolis).

Parents described experiencing doubt while using punishments. A 36-year-old mother of a 10-year-old child (large city) said that taking a phone away from a child is “not right”, because it “violates property”, but that is what parents have to do in order “to make it clear that the child is not allowed” to spend too much time on the smartphone.

Through ***rewards*** (money, purchases, praise, and access to digital devices) parents strove to encourage and consolidate the child’s successes in sports and studies, support initiatives in the classroom: “If you raise your hand more, you will have good grades.... And you can regulate your earnings…. You did a report, here’s an A and another 100 rubles. And I transfer that money to your card’” (44-year-old father of a 12-year-old child, megapolis).

These practices are aimed at subordinating children’s behavior, outlining a framework in which they can act independently of their parents, following established plans and rules.

***Clear, consistent requirements and deadlines*** suggested that a child should prioritize homework, know where and when to take a walk, obey traffic regulations, work hard and put in effort at school and sports and reduce the amount of screen time.

“He knows that at 20:00 he has to sit down for homework, and when it is already five to 20:00, I remind him: ‘It’s time to do homework’. That’s all, just at this time he finishes playing, and somewhere by 20:00 or 20:15 he is already sitting down” (38-year-old mother of a 10-year-old child, rural area).

There were two major areas where parents ***helped children with tasks and self-organization***: studying and initiating a new action. In studying, parents aimed to increase the child’s self-regulation (“sit down for lessons”, “it’s time to start”, “check”) and strengthen motivation (to find meaning in not very pleasant activities). Parents helped in performing complex tasks, where the children found it diffcult to cope on their own or asked for help.

Informants described ***helping children with making decisions*** about how much time to spend behind the screen and what smartphone game to choose, how to behave with other people, how to self-realize in hobbies. For this purpose, parents shared their own experience, explained common life patterns, encouraged children to imagine a certain situation and formulate their attitude to it, and showed techniques that facilitated making decisions.

***Conveying confidence in the child’s competence.*** Informants drew children’s attention to their progress and the skills they have gained in order to show that the child can handle a new task. This practice is one of the most effective for supporting autonomy, according to parents: “The most effective thing is when we tell him: ‘Look, you already know how to do this with someone else’. If you say that to him, some kind of electrical contact clicks inside and he’s like, ‘Yes, it’s true!’ ” (35-year-old mother of a 10-year-old child, large city).

In addition to those described in the literature, other practices of behavioral control and structure were discovered.

***Encouraging reflection and analysis of the situation.*** This technique was used when an uncomfortable circumstance needed to be adjusted in any way (whether a child couldn’t handle the amount of activity or whether the advantages of an action needed to be estimated). Parents motivated children to analyze forthcoming consequences to make a correct, thoughtful choice. This practice was presented in 13 interviews.

“When a child agrees to all extracurricular activities, and when it turns out that after school there is snowboarding, English, something else, and the child has no time left to socialize with peers. And then I say — well, let’s think about what is more important for you” (41-year-old father of a 13-year-old adolescent, millionaire city).

In our opinion, these practices can be interpreted in two ways. On the one hand, they encourage children to analyze a situation; they provide space for formulating a child’s opinions and attitudes to the situation. On the other hand, they are used to ensure that the child internally accepts and appropriates the parent’s point of view. Therefore, encouraging reflection may indirectly contribute to volitional functioning, but it directly stimulates independence.

***Guidance.*** This describes nudging in order to change unwanted non-autonomous behavior and is used when a child’s behavior does not conform to socially accepted, “correct” actions or to the image of a desirable lifestyle. Guidance could be characterized as rejection of the child’s point of view or as a structure aimed to promote autonomy by monitoring and rules (sometimes harshly) but in areas in which a parent wants to be present in the child’s life. Guidance appeared in 17 interviews.

“If you see that in the child’s activity there is a kind of result, expected, and expected is quite widely formulated, then it’s fine, let him do it himself. If you see that he is not coping... And what does it mean, is not coping? Doesn’t meet your expectations. That is, does not do his homework well, does not have friends, does not want to do anything in terms of hobbies, wants to just lie down, do nothing. So here it seems that we are already moving from autonomy to a kind of, well, well... Well, it’s coercion, but not coercion” (42-year-old father of a 6.5-year-old primary school child, megapolis).

***Explanation of patterns.*** This was described as explaining the outcomes of a certain behavior and is usually combined with behavioral control. Explanations were provided without encouraging any introspection, in contrast to encouraging reflection when a parent employed strategies to support a child’s mental process. Thirteen parents mentioned this practice.

“I put the phone away, and the child immediately said, ‘Mom, what?’ I explain: ‘You understand, it is bad for your health, for your eyes, and in general there is nothing good about being on [the phone]’ ” (45-year-old mother of a 13-year-old adolescent, small city).

***“Area of responsibility”.*** Parents frequently used the phrase “area of responsibility” to define some behaviors that children should routinely engage in on their own, free from adult supervision. It appeared in 10 interviews. The main “area of responsibility” was homework.

“I remember coming home and doing my homework, there were never any questions, no problems, nothing. That is, my parents were never interested in whether or not my homework was done; I had no other way, I always did everything. So I guess I’m still trying to instill in my child that his studies are his area of responsibility” (36-year-old mother of a 7-year-old child, small city).

Thus, behavioral control practices and structures in the behavior of Russian parents coexist with each other. Parents describe them as something that should help a child grow up to be independent. The types of behavioral control practices and structures are diverse and go beyond those described in the literature.

**Direct autonomy promotion and meeting other needs.** Statements demonstrating that parents strove to meet the child’s basic needs, from the point of view of self-determination theory, were found in 26 interviews. Parents tried to create conditions that would lead to a long-lasting result in the form of volitional functioning, but their actual behavior “here and now” implied support for children’s independent realization of goals set by their parents. Support for volitional functioning in real-life situations was only observed in certain situations.

***Providing a variety of choices*** was characterized by demonstrating various possibilities of behavior, spheres of activity. This practice was used for spending pocket money and choosing hobbies or additional education.

“I asked my daughter: ‘What do you think is important for you to do? We can stop going to the pool, in general, so that this situation does not occur anymore, when you get water in your ears and you have unpleasant sensations. Or you can continue with the classes, but exclude diving’ ” (48-year-old mother of a 12-year-old adolescent, megapolis).

***Explanation when choices are limited.*** Informants said that in cases of restrictions and prohibitions (night time communication via smartphone, timing and content of computer games, sweets consumption, getting a tattoo), they explained their rationale to their children. The father (39-year-old) of a 9-year-old child (megapolis) mentioned that as he set restrictions for PlayStation time to his child, he warned him about the dangers of excessive playing: “it will be harder, the brain will not be able to cope, you will feel bad”.

Parents talked about the importance of recognizing the child’s point of view in choosing additional education, hobbies, and when to eat. Parents found it necessary to be guided by their children’s opinion when building communication with them – speaking on equal terms as adults, apologizing if they feel they are wrong in a conflict.

“More often than not, we offer him things. He goes to the club of young engineers now ... he once went for a walk with his dad, saw this club, looked at it, evaluated it, was interested. But it was summer. In autumn we just reminded him, ‘Will you go?’ He said: ‘Yes, I will’. So, we kind of pushed the idea forward” (36-year-old mother of a 9-year-old child, megapolis).

***Providing care and enabling care for another.*** Parents showed concern for children’s physical and psychological well-being, accompanied them at late hours, paid attention to their feelings, and tried to help with problem solving via conversation or sharing their own experience. This applied above all to children’s relationships with their peers:

“I can explain why the boy acted this way [in response] to her actions. I tell her all the time that I, as a man, as a father, can give an explanation, an instruction, why it is the way it is. We need [to show] support, trust, our knowledge in relation to their life situations” (44-year-old father of a 12-year-old adolescent, megapolis).

Parents asked their children for help, where they themselves had difficulties, gave them the opportunity to take care of them, older or younger relatives, and pets:

“Doing my makeup is difficult for me. And then I had to go somewhere and I saw my daughter doing something, and I said: ‘Bunny, help me’. And she did my makeup and hair!” (41-year-old mother of a 12-year-old adolescent, millionaire city).

***Providing space.*** Parents were willing to reduce control and give space to children if they wanted to walk home from school, get extra classes, visit relatives, go out with friends. Digital monitoring and pre-teaching with a gradual decrease in monitoring was often an additional condition. Parents talked about letting children do their own lessons, choosing the order for themselves. This practice was described as a contribution to the child’s future development of autonomy.

“And if she has the initiative to do ‘the Environment’ [subject at school]. Well, then do it if you like” (36-year-old Mother of a 10-year-old child, large city).

Russian parents encourage children’s autonomy by providing space for independent action. They strive to expand the child’s ability to act autonomously and make decisions, encouraging his or her initiative, ensuring the right to choose, as well as showing care and warmth.

## Discussion

The aim of the study was to examine Russian parents of primary school children and early adolescents’ autonomy support and control practices and compare them with practices studied by foreign researchers.

Significant similarity of Russian parents’ practices of autonomy support and control with the types distinguished by foreign researchers was found. With the exception of “withholding love”, all practices described in the literature were present in the interviews. In a significant proportion of interviews, parents talked about practices of autonomy support (two types, but predominantly independence), structure and behavioral control, while the use of psychological control was mentioned less frequently. International studies show that regardless of the cultural context, psychological control negatively affects children’s development (Fang et al., 2021; Gao et al., 2021; Yan et al., 2020). Our study suggests that Russian children do not often face psychological control, which positively characterizes the conditions for the development of their independence. We assume that encouraging a child’s autonomy might be a socially approved behavior for Russian parents, making them more willing to talk about this practice, while psychological control might be perceived as a socially disapproved practice.

International studies demonstrate that the influence of behavioral control on a child’s autonomy is mediated by the cultural context (Lansford, 2022). Our data show that a feature of Russian parental practices is the wide representation and diversity of behavioral control and structure. Empirically, both of these behaviors are perceived as encouraging independence, which could be a culturally embedded pattern, common for collectivist and hierarchical countries (Marbell-Pierre et al., 2019). Parents strive to create a picture of the world that is understandable to the child, where there are stable requirements, reasonable restrictions and patterns of behavior. In such a world, as it seems to parents, children can make sense of their own behavior and regulate it, which will be their independence.

Manifestations of support for children’s autonomous behavior, initiative, and independence are as common as behavioral control and structure practices. Support for autonomy is much more common in our sample than psychological control. It can be concluded that Russian parents more often support autonomy than limit it. At the same time, they equally support children’s activity within existing structures and limitations (structure, behavioral control), and strive to satisfy the need for autonomy, competence, and belonging. Thus, the idea that Russian parents limit autonomy rather than support it is not justified.

We identified a variety of situations in which different practices were used. Psychological control practices were used when children attempted to reject school demands and expectations or were in a potentially dangerous setting for their mental and physical health. Behavioral control practices addressed time spent in digital environments, visiting dangerous places, substance use, school performance, and athletic success. Two types of autonomy support practices were used in areas such as independent mobility, studying, sports, doing chores, choosing additional education and hobbies, socializing with peers, and saving money. Practices were rarely presented in the narrative in a consistent and isolated way, which correlates with the paradigm of the absence of “pure” parenting styles (Smetana, 2017).

Several features emerged as parents talked about setting structure: Guidance, Explanation of Patterns and “Area of Responsibility”. These practices show parental intention not only to set norms, but also to get children to internalize them and act accordingly, as well as reflect on their actions.

In terms of autonomy support, the prevalence of one type over another was apparent in responses from parents of children of both age groups. Despite the fact that parents saw encouragement of children’s volitional functioning as something that directs their behavior, they mentioned it less vividly than encouragement of independence and articulated it in a more abstract manner. Parents promoted children’s volitional functioning when it reflected a parental perception of correct actions (usually in domains of independent mobility, socializing, engaging in academics and sports (which were usually suggested by parents), and rarely in online space).

Such phenomena can be explained in different ways. In one case, we can assume the orientation of parents’ actions to the needs of the children’s ages: the leading activities in primary school age and younger adolescence are learning and assimilation of social norms, respectively (Elkonin, 1989). In another case, it can be argued that there is a mismatch between what the parents aim to nurture in the child and what they have to nurture in reality, given different contexts or based on their own emotional condition (Martorell & Bugental, 2006). Lastly, it could demonstrate that for Russian culture, parenthood primarily involves leadership and taking responsibility for children’s well-being (Zakharova, 2008).

The situationality and variability of practices requires further reflection. On the one hand it can be interpreted as parental flexibility promoting autonomy, on the other hand as chaotic and inconsistent practices hindering it (Farkas & Grolnick, 2008; Soenens & Vansteenkiste, 2010).

The results can enrich the understanding of how Russian parents support the autonomy of their children. Direct opposition to autonomy is extremely rare. Most often, parents encourage independent behavior within the set norms and tasks, orienting the child in existing patterns and limitations. In some situations, parents encourage the initiative of the child, give him or her freedom of choice and action.

The following findings of this work may be useful for parents and teachers working with children:

- Certain practices undermine autonomy (accusation, induction of guilt) or limit it (deadlines, prohibitions);
- The situationality of parental autonomy support can lead to a child’s uncertainty about situation appropriate to be autonomous or not
- Child autonomy in different spheres can be formed heterochronously
- The flexibility of parents’ practices should be consistent with the practices of teachers.

## Conclusion

In this study we compared the practices stated by foreign researchers to be the most beneficial for autonomy support with those utilized on a daily basis by Russian parents of primary school children and early adolescents.

Specific features in the practices of autonomy support and control among Russian parents are:

- They often choose behaviors that should help their children become independent. They relatively rarely support volitional functioning;
- Behavioral control and structure practices and autonomy support practices are used with equal frequency;
- Psychological control practices are rarely used, so Russian parents cannot be called exclusively controlling.

Russian parents support their children’s autonomy in situations where children move within the agreed boundaries, does their lessons, chooses and participates in additional education and hobbies, socializes with peers, manages their money, and participates in household chores.

Russian parents limit their children’s autonomy when a child tries to visit dangerous places and engage in dangerous activities, violates school norms, is excessively involved in computer games or uses potentially harmful content, and also shows obstinacy and strong-willed behavior that does not coincide with the ideas of the parents. Parents forbid smoking and alcohol consumption.

We discovered the prevalence of support for independence in the answers of Russian parents. Volitional functioning support was less prominent and had some unique features: parents tended to promote initiative when it was regarded as “correct” or aligned with their own propositions.

We have gained evidence to suggest that parents may act in a variety of ways in different situations, and that there is both a consistent and contradictory mix of behaviors and beliefs. However, the question remains open as to the impact of this diversity on the child’s autonomy. The identified practices and their specific descriptions could form the basis for a questionnaire, guidelines, or an educational program for parents.

## Limitations

The main limitation of this work is the small sampling size. Unfortunately, the sample did not include respondents without higher education. Also, we did not directly address settings that influence parenting practices (family socioeconomic status, support system, presence of internal or externalizing problems in the child’s behavior, parental anxiety, and child-parent relationships) as well as emotional and cognitive components of child-parent relationships. Such significant factors as the practices of teachers, the representations of peers and the children themselves, the formation of skills, and the real experience of self-service, movement, study, communication, etc., remained outside the scope of the study. Additionally, due to the specifics of the sample, fathers’ practices may not have been sufficiently represented, which, however, opens the field for future work in this area: expanding the sample to include a larger proportion of men. Observing the daily behavior of parents could provide more objective information about autonomy promotion and control.
